# Commentary: Early supported discharge and transitional care management after stroke: a systematic review and meta-analysis

**DOI:** 10.3389/fneur.2026.1746857

**Published:** 2026-02-20

**Authors:** Ping Ma, Peijuan Zhang, Xuanling Zhou, Wenli Xu, Mingfei Yang, Xiuling Wei

**Affiliations:** 1 Department of Neurosurgery, Qinghai Provincial People's Hospital, Xining, Qinghai, China; 2 Graduate School, Qinghai University, Xining, Qinghai, China

**Keywords:** continuity of patient care, early supported discharge, meta-analysis, stroke, transitional care

## Introduction

We read with great interest the systematic review by Jee et al. (1). This study provided a comprehensive synthesis of the effectiveness of early supported discharge (ESD) and transitional care (TC) for patients after stroke, offering valuable insights for both research and clinical practice in this field. At the same time, we believe that several aspects related to the handling of heterogeneity and the interpretation of statistically non-significant pooled effects could be further clarified, which would help readers more accurately appraise the strengths and limitations of the evidence. The aim of this commentary is therefore to highlight key methodological considerations and issues of interpretation that may influence evidence synthesis and clinical inference, rather than simply restating the conclusions of the original review.

In the meta-analysis section, the authors first analyzed outcomes such as length of hospital stay, activities of daily living (ADL), modified Rankin Scale (mRS) scores, and caregiver burden using a fixed-effects model, and reported *I*^2^ values ranging from 59% to 90%, indicating substantial heterogeneity among the included studies. Switching to a random-effects model was therefore consistent with standard practice. However, the manuscript subsequently stated that “the random-effects model analysis did not show significant heterogeneity,” which might be misinterpreted as reflecting a potential methodological misunderstanding. It should be emphasized that, in meta-analysis, the choice of a random-effects model is typically driven by the presence of considerable heterogeneity and the need for a more robust pooled estimate, not because heterogeneity disappears after applying the model. The purpose of a random-effects model is to accommodate existing heterogeneity rather than to eliminate it.

In addition, Jee et al. reported a pooled random-effects estimate for length of stay of SMD = −0.13 (95% CI −0.31 to 0.04; *p* = 0.14). Since the confidence interval crosses zero and the *p*-value exceeds 0.05, this indicates that ESD or TC did not achieve a statistically significant reduction in length of hospital stay compared with usual care. In the “Results” section of the abstract, however, the authors stated that “ESD or TC could decrease the length of hospital stay more than the usual care.” Although this wording reflects a favorable direction of effect, which was clinically intuitive, explicitly stating that the difference was not statistically significant (*p* = 0.14) in the same place (or immediately thereafter) would help prevent readers from overemphasizing the direction of effect and would improve consistency between the abstract and the quantitative findings in the main text.

With respect to approaches for managing high heterogeneity, relevant examples can be found in the literature. For instance, Kim et al. conducted subgroup analyses to explore potential sources of heterogeneity in their study (2), and Michael et al. performed sensitivity analyses by excluding the largest or most heavily weighted study to assess the influence of a single study on the overall estimate (3). Such sensitivity and subgroup analyses can help explore factors influencing the effect size and identify potential sources of heterogeneity, for example according to stroke severity, intervention intensity, care setting, or other clinically relevant characteristics, allowing a more accurate interpretation of quantitative syntheses. If similar analyses were applied to outcomes with high heterogeneity in the present review, the interpretability and robustness of its conclusions would be further strengthened.

## Conclusion

Overall, the review by Jee et al. suggests that ESD and TC after stroke show a potentially favorable direction of effect for several outcomes; however, statistically significant and consistent benefits have not yet been demonstrated across all endpoints. Importantly, the use of a random-effects model does not resolve underlying clinical or methodological heterogeneity and should not be interpreted as justification for confidence in a single pooled estimate. Clearly indicating the non-significant nature of the main pooled estimates would enable readers to more accurately assess the true impact of ESD or TC models and enhance the transparency and clinical and policy relevance of the findings. Failure to appropriately account for and interpret substantial heterogeneity and non-significant pooled estimates may lead to overestimation of treatment effects and potentially inappropriate clinical interpretation and decision-making.
